# Modelling of Coupled Shrinkage and Creep in Multiphase Formulations for Hardening Concrete

**DOI:** 10.3390/ma12111745

**Published:** 2019-05-29

**Authors:** Peter Gamnitzer, Andreas Brugger, Martin Drexel, Günter Hofstetter

**Affiliations:** Unit of Strength of Materials and Structural Analysis, Institute of Basic Sciences in Engineering Sciences, Innsbruck University, Technikerstr. 13, A-6020 Innsbruck, Austria; Andreas.Brugger@uibk.ac.at (A.B.); Martin.Drexel@uibk.ac.at (M.D.); Guenter.Hofstetter@uibk.ac.at (G.H.)

**Keywords:** hardening concrete, hygro-thermo-chemo-mechanical modelling, shrinkage, creep

## Abstract

The durability and serviceability of concrete structures is influenced by both the early-age behavior of concrete as well as its long-term response in terms of shrinkage and creep. Hygro-thermo-chemo-mechanical models, as they are used in the present publication, offer the possibility to consistently model the behavior of concrete from the first hours to several years. However, shortcomings of the formulation based on effective stress, which is usually employed in such multiphase models, were identified. As a remedy, two alternative formulations with a different coupling of shrinkage and creep are proposed in the present publication. Both assume viscous flow creep to be driven by total stress instead of effective stress, while viscoelastic creep is driven either by total or effective stress. Therefore, in contrast to the formulation based on effective stress, they predict a limit value for shrinkage as observed in long-term drying shrinkage tests. Shrinkage parameters for the new formulations are calibrated based on drying shrinkage data obtained from thin slices. The calibration process is straightforward for the new formulations since they decouple shrinkage and viscous flow creep. The different formulations are compared using results from shrinkage tests on sealed and unsealed cylindrical specimens. Shrinkage strain predictions are significantly improved by the new formulations.

## 1. Introduction

Concrete structures such as bridges, tunnel linings, containments or fluid tanks are usually designed for a service life of several decades. It is therefore important to have reliable models for predicting the response of the loaded material for very long time periods. In that respect, the correct representation of shrinkage and creep phenomena is of utmost importance. However, cracking can be initiated already within the first days after casting. Thus, it is also important to have a profound understanding of the early-age processes, as cracking can seriously affect the serviceability of structures. The material properties of concrete are evolving due to cement hydration. Therefore, at early-age, the material will be most sensitive to external influences. Early-age loading can comprise mechanical loading, but also drying or thermal loading, which is often induced by self-heating due to chemical reactions. The thermal and hygral loads are coupled to mechanical loading in the sense that a spatial and temporal variation of temperature and pore humidity can cause volume reduction or expansion resulting in stresses in the material. In summary, a model is needed which properly accounts for thermal, hygral, chemical and mechanical phenomena and provides accurate predictions for both, the early-age and long-term response.

A pioneering approach addressing this challenge is the work on hygro-thermo-chemo-mechanical modelling of concrete by Gawin et al. [1,2]. The authors propose a fully coupled multiphase model accounting for hydration, shrinkage, and creep in an effective stress framework. Derived models have already been applied to many practical problems, for instance to concrete overlay and repair problems [3,4] or to sprayed concrete (shotcrete) linings [5]. The multiphase modelling approach for concrete still receives a lot of attention in the research community. Two examples of very recent developments in that context are multiphase models with an improved modelling of the evolution of the solid phase [6] and multiphase models with a more accurate prediction of early-age autogenous shrinkage based on the application of a porosity-dependent desorption isotherm [7].

However, shortcomings of the original effective stress multiphase approach have also been identified, and the need for further improvements has already been stated in [7]. Although drying shrinkage was predicted well by the effective stress approach during the first year, the predicted response turned out to be inappropriate for longer drying periods. Hence, the focus of the present publication is to propose remedies for this shortcoming. Compared to the results obtained in [7], the methods developed in the present work will provide (i) more realistic long term shrinkage predictions, (ii) a decoupling of shrinkage and viscous flow creep (and vice versa) and therefore (iii) a simplified calibration procedure for the shrinkage model. Due to the proposed improvements it is now possible to provide a further validation of the multiphase model for test durations up to two years, instead of one year as it was done for the original effective stress multiphase approach in [7].

The publication [7] provides a comprehensive calibration of all model parameters of the original form of the coupled model.

This calibration is based on a large set of experiments for the concrete mixture summarized in Table 1, including calorimetry tests, tests for age-dependent mechanical properties, tests for determining the hygral properties, different shrinkage tests, as well as compressive creep tests [8,9,10,11]. For this reason, it forms the basis for the developments in the present paper. It is nevertheless important to identify which of the previously calibrated parameters remain unchanged for the methods developed in the present work.

The calibration of the long-term creep parameters in [7] was based on creep compliances, and is therefore independent of the shrinkage formulation. This allows investigating different coupling strategies between shrinkage and creep in the present paper using the same set of creep parameters.

Accurate modelling of the evolution of mechanical properties (Young’s modulus and compressive strength), cement hydration, and hygral properties was proven by the results presented in [7]. These results are not affected by the changes in the model, introduced in the following, and will therefore not be repeated in the present publication. The simulations in the present paper are restricted to experiments on the specific concrete mixture used in [8,9,10,11], because it is the only mixture known to the authors for which a complete set of multiphase-model parameters is available.

The original formulation of the hygro-thermo-chemo-mechanical multiphase model [1,2] assumes a single effective stress variable governing shrinkage and creep of the material. The apparent limitations of using a single effective stress variable for modelling partially saturated materials have long been known in the geotechnical engineering community. For describing inelastic deformation of partially saturated soils it is a well-established approach to use a second stress state variable in the mechanical constitutive model, combining a Bishop-type effective stress or a net stress with matric suction as a second stress state variable [12,13,14]. An overview on effective stress approaches in the geomechanical context is provided in [15]. From this perspective, the model proposed in the present paper mimics this approach by introducing a second stress state variable in the multiphase creep formulation, finally resulting in a so-called mixed stress formulation. This formulation is introduced herein, and it is compared to a total stress and to the traditional effective stress formulation as well.

The paper is structured as follows. Section 2 provides an overview of the governing equations of the coupled problem together with the respective parameter values obtained by the calibration in [7]. The shrinkage and creep models are presented in Section 3, the original effective stress approach is reviewed, and the mixed and total stress formulations are introduced. A comparison of the three coupled shrinkage and creep formulations is provided in Section 4. The characteristics of the three approaches are highlighted by simulations of drying tests on thin concrete slices and by simulations of cylindrical specimens. Final conclusions are drawn in Section 5.

## 2. Governing Equations

In the following, the governing equations of the fully coupled multiphase model are reviewed briefly and the results of parameter calibrations, presented in [7], are summarized. The intention of this summary is (i) to clarify which parts of the model, and which parameters, are unaffected by the model improvements that will be proposed later, and (ii) to provide, in a condensed way, all information required for the respective numerical simulations.

The hygro-thermo-chemo-mechanical model, used in the present paper, is based on three primary unknowns: Displacement u, capillary pressure $p^{c}$, and temperature *T*. Instead of including the pore gas pressure $p^{g}$ as a further primary unknown, a passive gas phase is assumed which means that $p^{g}$ is assumed to be constant and equal to the atmospheric pressure $p^{\mathrm{atm}}$. The chosen sequence of the following equations is motivated by starting with deriving the dependent thermodynamic state variables from these primary unknowns. Further equations are then added in a step-by-step procedure, providing the basis for the investigations in the subsequent section.

### 2.1. Derived Thermodynamic State Variables

Pore water pressure $p^{w}$, relative pore humidity φ, vapor pressure $p^{\mathrm{gw}}$ and dry air pressure $p^{\mathrm{ga}}$ are derived quantities according to the following equations taken from [16]. The water pressure equals the difference between the pore gas pressure and the capillary pressure, viz.

(1) $$p^{w}=p^{g}-p^{c}.$$

The relation between $p^{c}$ and the relative pore humidity φ, which is the ratio between vapor pressure and the temperature-dependent vapor pressure at full saturation $p_{\mathrm{sat}}^{\mathrm{gw}}$, is given by the Kelvin-Laplace law

(2) $$\varphi =\frac{p^{\mathrm{gw}}}{p_{\mathrm{sat}}^{\mathrm{gw}}}=\exp\left(-\frac{p^{c}\;\cdot \;M_{w}}{\rho_{w}\;\cdot \;R\;\cdot \;T}\right)\;,$$

with the molar mass of water $M_{w}$ and the universal gas constant *R*. The density of liquid water $\rho_{w}$ is assumed to be constant. The temperature dependence of the vapor pressure at full saturation is governed by the Clausius-Clapeyron equation

(3) $$p_{\mathrm{sat}}^{\mathrm{gw}}=p_{\mathrm{sat},0}^{\mathrm{gw}}\;\cdot \;\exp\left(-\frac{\Delta H_{\mathrm{vap}}\;\cdot \;M_{w}}{R}\;\cdot \;\left(\frac{1}{T}-\frac{1}{293K}\right)\right)\;.$$

The enthalpy of evaporation $\Delta H_{\mathrm{vap}}$ is assumed to be constant, $p_{\mathrm{sat},0}^{\mathrm{gw}}$ denotes the vapor pressure at full saturation and reference temperature. Dry air pressure $p^{\mathrm{ga}}$ is obtained from Dalton’s law as the difference between gas pressure and vapor pressure $p^{\mathrm{gw}}$

(4) $$p^{\mathrm{ga}}=p^{g}-p^{\mathrm{gw}}\;.$$

The densities of water vapor $\rho_{\mathrm{gw}}$ and dry air $\rho_{\mathrm{ga}}$ are computed based on the equation of state of an ideal gas, viz.

(5) $$\rho_{\mathrm{gw}}=\frac{M_{w}\;\cdot \;p^{\mathrm{gw}}}{R\;\cdot \;T}\;\mathrm{and}\;\rho_{\mathrm{ga}}=\frac{M_{a}\;\cdot \;p^{\mathrm{ga}}}{R\;\cdot \;T}\;.$$

The parameters in Equations (2) to (5) are summarized in Table 2.

### 2.2. Hydration Model and Porosity Evolution

The complex process of cement hydration is lumped into a single Arrhenius-type reaction model, a simplified approach which is widespread in multiphase modelling [1,3,4,6]. The state of hydration is quantified by a single dimensionless parameter, the normalized degree of hydration Γ, which varies between zero and one. It is given, following [17], as the ratio of chemically bound water per unit volume at time *t*, $\Delta m_{w}\left(t\right)$, and the ultimate amount of chemically bound water per unit volume $\Delta m_{w}^{\infty }$:

(6) $$\Gamma =\frac{\Delta m_{w}\left(t\right)}{\Delta m_{w}^{\infty }}\;.$$

Its evolution is described by [1,18]:

(7) $$\dot{\Gamma }=\left[A_{1}\;\cdot \;\left(\frac{A_{2}}{\kappa_{\infty }^{w/c}}+\kappa_{\infty }^{w/c}\;\cdot \;\Gamma \right)\;\cdot \;\left(1-\Gamma \right)\;\cdot \;\exp\left(-\bar{\eta }\;\cdot \;\Gamma \right)\right]\;\cdot \;\frac{1}{1+625\;\cdot \;{\left(1-\varphi \right)}^{4}}\;\cdot \;\exp\left(-\frac{E_{a}}{R\;\cdot \;T}\right)\;.$$

The hydration rate is affected by (i) the current state of hydration Γ, described by the term in square brackets in (7) with parameters $A_{1}$, $A_{2}$, $\bar{\eta }$ and $\kappa_{\infty }^{w/c}$, (ii) the relative pore humidity φ, and (iii) the temperature, described by the last term with the parameter $E_{a}$ denoting the activation energy. For fully coupled hygro-thermo-chemo-mechanical models an initial value $\Gamma_{\mathrm{init}}$ is used for the normalized degree of hydration. It corresponds to the degree of hydration at which the hardening concrete is assumed to solidify [3,19]. This state is reached for the present concrete mixture approximately 8 h after the water was added during the mixing procedure. All subsequent computations are started at this concrete age. Values for the above-mentioned parameters were calibrated for the concrete mixture of Table 1 in [7]. They are listed in Table 3.

As in [1], concrete is treated in the present paper as a porous three-phase medium consisting of two fluid phases, gas and liquid water, which are contained in the pore space of a homogenous solid phase of constant density $\rho_{s}$. The porosity *n* of the three-phase medium is quantified by the ratio between void volume and total volume of a representative volume element. Hydration is assumed to result in a linear decrease of porosity [1,3]:

(8) $$n=n_{\infty }+A_{n}\;\cdot \;\left(1-\Gamma \right)\;.$$

Values for the final porosity $n_{\infty }$ and the parameter $A_{n}$ for the present concrete mixture were also calibrated in [7]. They are listed in Table 3.

### 2.3. Desorption Isotherm

The evaporable water content $w_{e}$ is the mass of evaporable water per unit volume of the porous material [21]. It can be expressed in the framework of the theory of porous media as the sum of the liquid water content and the water vapor content:

(9) $$w_{e}=\rho_{w}\;\cdot \;n\;\cdot \;S_{w}+\rho_{\mathrm{gw}}\;\cdot \;n\;\cdot \;\left(1-S_{w}\right)\;.$$

The ratio between water volume and pore space volume in a representative volume element is $S_{w}$, the degree of water saturation. Except for extreme conditions, the liquid water content is the dominating part due to the density ratio between liquid water and water vapor, i.e., most of the water mass is present in liquid form. In the present approach, the desorption isotherm is modelled by assuming a moisture retention function

(10) $$S_{w}\left(p^{c},n\right)={\left[1+{\left(\frac{p^{c}}{p_{b}^{c}\left(n\right)}\;\cdot \;\exp\left(D\;\cdot \;\frac{p^{c}}{p_{b}^{c}\left(n_{\infty }\right)}\right)\right)}^{n_{\mathrm{Sw}}}\right]}^{-m_{\mathrm{Sw}}}$$

in terms of capillary pressure and porosity as proposed in [7]. The parameters $n_{\mathrm{Sw}}$, $m_{\mathrm{Sw}}$, and *D* are dimensionless fitting parameters. The porosity dependence is included via a porosity-dependent air entry value

(11) $$p_{b}^{c}\left(n\right)=p_{b}^{c}\left(n_{\infty }\right)\;\cdot \;{\left[\frac{\left(1-n\right)}{n}\;\cdot \;\frac{n_{\infty }}{\left(1-n_{\infty }\right)}\right]}^{\psi },$$

which is defined using the air entry value at full hydration $p_{b}^{c}\left(n_{\infty }\right)$ and a power-law exponent ψ. The dependence of $S_{w}$ on capillary pressure can be converted to a dependence on relative humidity using the Kelvin-Laplace law (2). The dependence of $S_{w}$ on porosity is included for considering the dependence of the desorption isotherm on the microstructure and pore size distribution of the porous medium. The latter is affected by the ongoing hydration process, see [3,22] for similar approaches. Further information on the moisture retention function (10), together with three-dimensional visualizations of the corresponding desorption isotherm surface, are available in [7].

For ψ=D=0, the moisture retention function defined by (10) and (11) is equivalent to the moisture retention curve by van Genuchten [23]. For D=0, the moisture retention function proposed by Gallipoli et al. [24] for soils is recovered. The parameters in (10) and (11) were calibrated for the concrete mixture of Table 1 in [7]. The respective values are listed in Table 4.

### 2.4. Balance Equations

In this section the balance equations for the fully coupled hygro-thermo-chemo-mechanical model [1,16,25] are summarized. Effects of gravity are neglected, which is a reasonable assumption for the specimens investigated in the following.

#### 2.4.1. Balance of Momentum for the Multiphase Continuum

Quasi-static equilibrium of the multiphase continuum can be stated in terms of the total stress σ as

(12) ∇∘σ=0.

The total stress is related to strain, capillary pressure, and temperature according to a constitutive relationship accounting for short-term and long-term behavior.

#### 2.4.2. Balances of Mass for the Solid Phase and the Water Phase

The mass balance for the solid phase reads

(13) $$\frac{d}{dt}\left(\left(1-n\right)\;\cdot \;\rho_{s}\right)+\left(1-n\right)\;\cdot \;\rho_{s}\;\cdot \;\left(\nabla \circ \dot{u}\right)=\dot{\Gamma }\;\cdot \;\Delta m_{w}^{\infty }\;.$$

The density of the solid phase $\rho_{s}$ is assumed to be independent of the degree of hydration and the deformation. A change in the solid phase content $\left(1-n\right)\;\cdot \;\rho_{s}$ of the porous medium is therefore solely governed by a change in porosity.

The mass balance of water reads

(14) $$\begin{array}{c} \frac{d}{dt}\left(w_{e}\right)+w_{e}\;\cdot \;\left(\nabla \circ \dot{u}\right)+\nabla \circ \left(\frac{\rho_{w}k_{w}^{\mathrm{rel}}K}{\mu_{w}}\;\cdot \;\nabla p^{c}\right)-\nabla \circ \left(\rho_{g}D_{g}^{\mathrm{gw}}\;\cdot \;\nabla \left(\frac{\rho_{\mathrm{gw}}}{\rho_{g}}\right)\right)=-\dot{\Gamma }\;\cdot \;\Delta m_{w}^{\infty }\;. \end{array}$$

In (14), water transport is modelled using Darcy’s law for liquid water and Fick’s diffusion law for water vapor. Chemically bound water is considered as a part of the solid skeleton. Therefore, the exchange term $\dot{\Gamma }\;\cdot \;\Delta m_{w}^{\infty }$ due to cement hydration is considered as a source term in (13) while it acts as a sink term in (14). The density of the gas phase $\rho_{g}$ is the sum of water vapor density $\rho_{\mathrm{gw}}$ and dry air density $\rho_{\mathrm{ga}}$. The dynamic viscosity of water $\mu_{w}$ is assumed constant. As in Gawin et al. [1], the permeability of concrete *K* depends on the degree of hydration according to

(15) $$K\left(\Gamma \right)=K_{\infty }\;\cdot \;10^{A_{\mathrm{perm}}\left(1-\Gamma \right)}\;.$$

Parameters are the dimensionless value $A_{\mathrm{perm}}$ and the asymptotic intrinsic permeability $K_{\infty }$. The relative water permeability in a partially saturated state is assumed to be of van Genuchten-Mualem type [23,26]

(16) $$k_{w}^{\mathrm{rel}}\left(S_{w}\right)=\sqrt{S_{w}}\;\cdot \;{\left[1-{\left(1-S_{w}^{1/m_{\mathrm{kw}}}\right)}^{m_{\mathrm{kw}}}\right]}^{2}\;.$$

The relation contains a single dimensionless parameter $m_{\mathrm{kw}}$. The diffusion coefficient $D_{g}^{\mathrm{gw}}$ for water vapor in the air contained in the pore space is a saturation-, porosity-, and temperature-dependent quantity [4,27]:

(17) $$D_{g}^{\mathrm{gw}}=D_{g,0}^{\mathrm{gw}}\;\cdot \;{\left(\frac{T}{273K}\right)}^{5/3}\;\cdot \;f_{S}\left(n,S_{w}\right),\;f_{S}\left(n,S_{w}\right)=n^{a_{\mathrm{fS}}}\;\cdot \;{\left(1-S_{w}\right)}^{b_{\mathrm{fS}}}\;.$$

The coefficient of diffusion for water vapor in the air at reference temperature is $D_{g,0}^{\mathrm{gw}}$. It is reduced for diffusion inside a porous medium by the resistance factor $f_{S}$ which depends on the two parameters $a_{\mathrm{fS}}$ and $b_{\mathrm{fS}}$. Boundary conditions for the simulation of drying processes are modelled following the approach of Sciumé et al. [3] by assuming the water mass flux $q_{W}$ across the drying surface as proportional to the difference $\Delta p^{c}$ of the capillary pressure on the surface and the capillary pressure resulting from the ambient humidity at the ambient temperature of 293 K, viz.

(18) $$q_{W}=\beta_{W}\Delta p^{c}\;\cdot \;n$$

with n denoting the unit normal vector to the surface at the respective point. The transport parameters were calibrated for the concrete mixture of Table 1 in [7]. They are summarized in Table 5.

#### 2.4.3. Balance of Enthalpy for the Multiphase Mixture

The effective heat capacity per unit volume of partially saturated concrete is

(19) $$C_{p}^{\mathrm{eff}}\rho^{\mathrm{eff}}=\left(1-n\right)\;\cdot \;C_{p}^{s}\rho_{s}+n\;\cdot \;\left[C_{p}^{w}\left(S_{w}\rho_{w}+\left(1-S_{w}\right)\rho_{\mathrm{gw}}\right)+C_{p}^{\mathrm{ga}}\left(\left(1-S_{w}\right)\rho_{\mathrm{ga}}\right)\right]\;,$$

depending on the specific heat capacities of water $C_{p}^{w}$, dry air $C_{p}^{\mathrm{ga}}$, and the solid phase $C_{p}^{s}$, respectively. It appears in the storage term of the balance of enthalpy for the multiphase mixture

(20) $$\begin{array}{c} C_{p}^{\mathrm{eff}}\rho^{\mathrm{eff}}\;\cdot \;\frac{d}{dt}T+\left(C_{p}^{w}\rho_{w}\left(\frac{k_{w}^{\mathrm{rel}}K}{\mu_{w}}\;\cdot \;\nabla p^{c}\right)\right)\circ \nabla T-\nabla \circ \left(\lambda_{\mathrm{eff}}\nabla T\right)=-\Delta H_{\mathrm{vap}}\;\cdot \;{\dot{m}}_{\mathrm{vap}}+\dot{\Gamma }\;\cdot \;Q_{\infty }\;. \end{array}$$

The transport of heat is dominated by heat conduction which is governed by the parameter $\lambda_{\mathrm{eff}}$. Heat is generated by hydration. The value $Q_{\infty }$ denotes the released heat of hydration per unit volume. Evaporation of water causes cooling. The respective decrease in temperature is proportional to the mass rate of vaporizing water

(21) $$\begin{array}{c} {\dot{m}}_{\mathrm{vap}}=-\left[\frac{d}{dt}\left(n\rho_{w}S_{w}\right)+n\rho_{w}S_{w}\;\cdot \;\left(\nabla \circ \dot{u}\right)+\nabla \circ \left(\frac{\rho_{w}k_{w}^{\mathrm{rel}}K}{\mu_{w}}\;\cdot \;\nabla p^{c}\right)+\dot{\Gamma }\;\cdot \;\Delta m_{w}^{\infty }\right]\;. \end{array}$$

A convective heat transfer condition is used in the simulations presented in this paper, assuming the convective heat flux in the direction of the unit normal n to be proportional to the difference between surface and ambient temperature ΔT:

(22) $$q_{T}=\beta_{T}\Delta T\;\cdot \;n\;.$$

The proportionality parameter is the convective heat transfer coefficient $\beta_{T}$. The parameters for the balance of enthalpy for the concrete mixture of Table 1 are summarized in Table 6.

## 3. Shrinkage and Creep Models

### 3.1. Stress Variables in Multiphase Models and Creep Driving Stress

A decomposition of the total stress σ in an effective stress $\sigma^{\mathrm{eff}}$ (acting on the solid skeleton) and a hydrostatic, pore-pressure-induced stress $\sigma^{\mathrm{pore}}$ (transmitted by the pore fluids) is assumed:

(23) $$\sigma^{\mathrm{eff}}=\sigma -\sigma^{\mathrm{pore}}\;.$$

The pore-pressure-induced part is modelled as a function of capillary pressure and saturation according to the generalized Bishop-parameter formulation

(24) $$\sigma^{\mathrm{pore}}=1\;\cdot \;\alpha_{\mathrm{Biot}}\;\cdot \;\chi \left(S_{w}\right)\;\cdot \;p^{c}\;.$$

As in [4,7], for instance, the generalized Bishop parameter $\alpha_{\mathrm{Biot}}\;\cdot \;\chi$ is approximated by a linear function of saturation:

(25) $$\alpha_{\mathrm{Biot}}\;\cdot \;\chi \left(S_{w}\right)=a_{\chi }\;\cdot \;S_{w}-b_{\chi }\;.$$

Compressibility of the solid phase (in the sense of a Biot coefficient factor $\alpha_{\mathrm{Biot}}$, see [16]) can be accounted for by selecting appropriate values for the constants $a_{\chi }$ and $b_{\chi }$.

The pore-pressure induced part exerts a pressure on the solid matrix in the partially saturated regime. It is considered as the source of drying shrinkage deformation. Depending on the actual choice of the shrinkage formulation, the resulting drying shrinkage strain can comprise an elastic strain as well as the strain $\varepsilon^{\mathrm{cve}}$ induced by viscoelastic creep and the strain $\varepsilon^{\mathrm{cf}}$ originating from viscous flow creep.

The evolution of the creep strain is stress-driven. However, in the multiphase-context, different stress variables are available for modelling the evolution of the viscoelastic and viscous flow creep strain. The stress which is actually used in the viscoelastic and viscous flow creep formulation will be referred to as the creep-driving stress $\sigma^{\mathrm{drive}}$ in the following. Therefore, for the stress driving the viscoelastic creep strain evolution one can use either

(26) $$\sigma^{\mathrm{drive},\;\mathrm{ve}}=\sigma^{\mathrm{eff}}\;\mathrm{or}\;\sigma^{\mathrm{drive},\;\mathrm{ve}}=\sigma \;,$$

and for the stress driving the viscous flow creep strain evolution one can use either

(27) $$\sigma^{\mathrm{drive},\;f}=\sigma^{\mathrm{eff}}\;\mathrm{or}\;\sigma^{\mathrm{drive},\;f}=\sigma \;.$$

### 3.2. Evolution of the Creep Strain

The creep strain rate is additively decomposed in the viscoelastic and the viscous flow part, ${\dot{\varepsilon }}^{\mathrm{cve}}$ and ${\dot{\varepsilon }}^{\mathrm{cf}}$, respectively:

(28) $${\dot{\varepsilon }}^{\mathrm{cr}}={\dot{\varepsilon }}^{\mathrm{cve}}+{\dot{\varepsilon }}^{\mathrm{cf}}\;.$$

The viscoelastic part is modelled by associating the hydration process with the solidification of a non-aging viscoelastic material [2,28,29]. The viscous flow part is modelled by a microprestress approach [30,31]. The range of application for the viscoelastic and viscous flow creep models described below is limited to the linear creep regime.

#### 3.2.1. Viscoelastic Creep Strain Rate

The viscoelastic part of the creep strain rate according to the solidification theory of concrete creep reads:

(29) $${\dot{\varepsilon }}^{\mathrm{cve}}=\frac{1}{\Gamma \left(t\right)}\int_{0}^{t}\frac{d\Phi }{dt}\left(t-t^{\prime }\right)G{\dot{\sigma }}^{\mathrm{drive},\;\mathrm{ve}}\left(t^{\prime }\right)\;dt^{\prime }\;.$$

The matrix G is the elastic compliance matrix of Hooke’s law for a unit elastic modulus and Poisson’s ratio ν:

(30) $$G=\left[\begin{array}{cccccc} 1 & -\nu & -\nu & 0 & 0 & 0\\ -\nu & 1 & -\nu & 0 & 0 & 0\\ -\nu & -\nu & 1 & 0 & 0 & 0\\ 0 & 0 & 0 & 2\left(1+\nu \right) & 0 & 0\\ 0 & 0 & 0 & 0 & 2\left(1+\nu \right) & 0\\ 0 & 0 & 0 & 0 & 0 & 2\left(1+\nu \right) \end{array}\right]$$

The matrix (30) extends the uniaxial creep law to a multiaxial creep law [30]. The age-independent microscopic creep compliance function of the solidified matter Φ is given in terms of a compliance parameter $q_{2}$ and a power-law exponent $n^{\mathrm{Kelvin}}$ [28]. It is approximated by a truncated Dirichlet series [32] of *N* Kelvin chain units with a minimal retardation time $\tau_{0}^{\mathrm{Kelvin}}$ and a retardation time ratio $\tau_{i+1}^{\mathrm{Kelvin}}/\tau_{i}^{\mathrm{Kelvin}}=10$:

(31) $$\Phi \left(t-t^{\prime }\right)=q_{2}\ln\left(1+{\left(\frac{t-t^{\prime }}{1d}\right)}^{n^{\mathrm{Kelvin}}}\right)\approx \frac{1}{E_{0}^{\mathrm{Kelvin}}}+\sum_{i=1}^{N}\frac{1}{E_{i}^{\mathrm{Kelvin}}}\;\cdot \;\left(1-\exp\left(-\frac{t-t^{\prime }}{\tau_{i}^{\mathrm{Kelvin}}}\right)\right)\;.$$

The term $1/E_{0}^{\mathrm{Kelvin}}$ represents the contribution of the part of the spectrum with very short retardation times. It can be neglected if the first retardation time is sufficiently small [21]. The compliances of the Kelvin units are given as:

(32) $$\frac{1}{E_{i}^{\mathrm{Kelvin}}}=q_{2}\;\cdot \;\ln\left(10\right)\;\cdot \;n^{\mathrm{Kelvin}}\;\cdot \;\left(1-n^{\mathrm{Kelvin}}\right)\;\cdot \;\frac{3{\left(\tau_{i}^{\mathrm{Kelvin}}\right)}^{n^{\mathrm{Kelvin}}}}{1+3{\left(\tau_{i}^{\mathrm{Kelvin}}\right)}^{n^{\mathrm{Kelvin}}}}\;.$$

Values of the viscoelastic creep parameters for the concrete mixture of Table 1 were calibrated in [7] from Young’s modulus tests. The obtained values are summarized in Table 7. These tests for determining the elastic modulus are of short duration and were performed at constant temperature. Therefore, they are not affected by temperature or drying shrinkage. Hence, these values are independent of the actual choice of the creep driving stress variable, which is of major importance for the following investigation.

#### 3.2.2. Viscous Flow Creep

For the viscous flow part of the creep law a linear relation between the viscous flow creep strain rate ${\dot{\varepsilon }}^{\mathrm{cf}}$ and the respective creep-driving stress $\sigma^{\mathrm{drive},\;f}$ is assumed:

(33) $${\dot{\varepsilon }}^{\mathrm{cf}}=\frac{1}{\eta_{S}}\;\cdot \;G\sigma^{\mathrm{drive},\;f}\;.$$

The viscosity $\eta_{S}$ is dependent on time and pore humidity history. This dependency is modelled in the framework of the microprestress theory [30,31,33] in terms of a temporally decaying microprestress *S*:

(34) $$\eta_{S}=\frac{1}{c_{0}q_{4}S}\;.$$

Starting from an initial value $S_{0}$, the evolution of this microprestress is governed by the evolution law

(35) $$\dot{S}+c_{0}S^{2}=-c_{1}\;\cdot \;\dot{\left(\ln\varphi \right)}$$

with the three material parameters $q_{4}$, $c_{0}$, and $c_{1}$. They were calibrated in [7] based on creep compliance data, which was obtained as a load-normalized difference between total strain (obtained from loaded specimens) and shrinkage strain (obtained from companion shrinkage tests).

The respective values are listed in Table 7. It is important to emphasize that shrinkage does not affect the calibration of the viscous flow creep parameters since it was performed by means of the creep compliance functions only [7]. Therefore, the viscous flow creep parameters do not depend on the choice of the creep driving stress variable and can be used for all formulations proposed in the present paper.

### 3.3. Stress-Strain Relationship and Effective Young’s Modulus

The microprestress solidification approach results in a quasi-elastic stress-strain relationship with an incremental stiffness [31]. It relates the total stress increment Δσ to the increments of total strain Δε, creep strain $\Delta \varepsilon^{\mathrm{cr}}$, thermal strain $\Delta \varepsilon^{\mathrm{th}}$, and shrinkage strain $\Delta \varepsilon^{\mathrm{sh}}$:

(36) $$\Delta \sigma =E_{\mathrm{qe}}\;\cdot \;G^{-1}\;\cdot \;\left(\Delta \varepsilon -\Delta \varepsilon^{\mathrm{cr}}-\Delta \varepsilon^{\mathrm{th}}-\Delta \varepsilon^{\mathrm{sh}}\right)\;.$$

The incremental stiffness $E_{\mathrm{qe}}$ is dependent on the time increment as well as on the degree of hydration (and therefore implicitly on time):

(37) $$E_{\mathrm{qe}}=E_{\mathrm{qe}}\left(t,\Delta t\right)={\left(1/E_{\mathrm{asym}}+1/E_{\mathrm{ve}}\left(\Delta t\right)\right)}^{-1}\;.$$

As proposed in [2], it contains a contribution related to a hydration-dependent asymptotic elastic compliance

(38) $$\frac{1}{E_{\mathrm{asym}}}=\frac{1}{E_{\mathrm{asym},\;\infty }}\;\cdot \;{\left(\frac{\Gamma -\Gamma_{\mathrm{init}}}{1-\Gamma_{\mathrm{init}}}\right)}^{-b_{E}}$$

as well as a time-increment and hydration-dependent viscoelastic part which is given as:

(39) $$\frac{1}{E_{\mathrm{ve}}\left(\Delta t\right)}=\frac{1}{\Gamma }\left(\frac{1}{E_{0}^{\mathrm{Kelvin}}}+\sum_{i=1}^{N}\frac{1}{E_{i}^{\mathrm{Kelvin}}}\;\cdot \;\left(1-\left(1-\exp\left(-\frac{\Delta t}{\tau_{i}^{\mathrm{Kelvin}}}\right)\;\cdot \;\frac{\tau_{i}^{\mathrm{Kelvin}}}{\Delta t}\right)\right)\right)\;.$$

The asymptotic elastic compliance defines the relationship between the stress driving the viscoelastic creep $\sigma^{\mathrm{drive},\;\mathrm{ve}}$ and the elastic strain $\varepsilon^{\mathrm{asym}}$, i.e., the instantaneous part of the material response. Values for the two material parameters $E_{\mathrm{asym},\;\infty }$ and $b_{E}$ calibrated for the concrete mixture of Table 1 in [7] are listed in Table 8 together with the thermal expansion coefficient $\alpha_{T}$ which governs the evolution of the thermal strain according to

(40) $${\dot{\varepsilon }}^{\mathrm{th}}=\alpha_{T}\dot{T}\;\cdot \;1\;.$$

### 3.4. Coupled Multiphase Shrinkage and Creep Formulations

Three variants of a coupled multiphase shrinkage and creep formulation are investigated in the following. For all of them, the change in shrinkage strain is governed by the change in pore fluid stress (24). The formulations differ by the coupling between the shrinkage and creep formulations which results from the use of different creep driving stresses in (29) and (33). The three variants will be introduced below. It is emphasized once more that they all work with the same creep parameters, which were obtained in [7] solely based on creep compliances. Only the values for the parameters $a_{\chi }$ and $b_{\chi }$, defining the dependency of the Bishop parameter on saturation according to (25), are different.

#### 3.4.1. Original Multiphase Shrinkage and Creep Formulation Driven by Effective Stress

**Figure 1:**
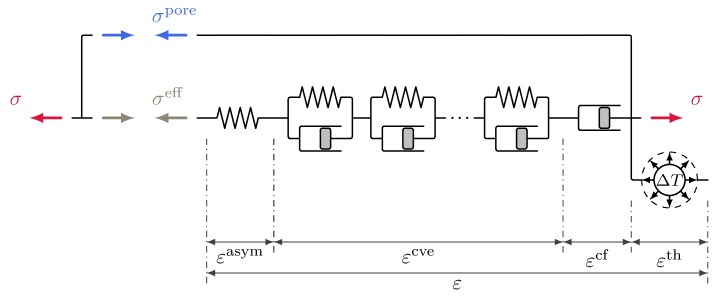
Schematic representation of the multiphase shrinkage and creep formulation driven by a single stress variable, the effective stress.

The first variant investigated here is the original coupled multiphase shrinkage and creep formulation proposed in [1,2]. It is characterized by the choice

(41) $$\sigma^{\mathrm{drive},\;\mathrm{ve}}=\sigma^{\mathrm{drive},\;f}=\sigma^{\mathrm{eff}},$$

i.e., by assuming both, viscoelastic and viscous flow creep to be driven by the effective stress. This approach is illustrated schematically in Figure 1. The quasi-elastic stress-strain relationship (36) can be stated for this approach in the form

(42) $$\Delta \sigma =E_{\mathrm{qe}}\;\cdot \;G^{-1}\;\cdot \;\left(\Delta \varepsilon -\Delta \varepsilon^{\mathrm{cr}}-\Delta \varepsilon^{\mathrm{th}}-\Delta \varepsilon^{\mathrm{sh}}\right)\;\mathrm{with}\;\Delta \varepsilon^{\mathrm{sh}}=-1\;\cdot \;\frac{\Delta \left(\alpha_{\mathrm{Biot}}\;\cdot \;\chi \left(S_{w}\right)\;\cdot \;p^{c}\right)}{3K_{\mathrm{qe}}}$$

with the rate and hydration dependent effective elastic-viscoelastic bulk modulus

(43) $$K_{\mathrm{qe}}=\frac{E_{\mathrm{qe}}}{3\left(1-2\nu \right)}\;.$$

Relation (42) can be rewritten in terms of the effective stress as

(44) $$\Delta \sigma^{\mathrm{eff}}=E_{\mathrm{qe}}\;\cdot \;G^{-1}\;\cdot \;\left(\Delta \varepsilon -\Delta \varepsilon^{\mathrm{cr}}-\Delta \varepsilon^{\mathrm{th}}\right)\;.$$

At first view, this seems to be the most straightforward approach of coupling shrinkage and creep. However, the fact that the pore humidity evolution, and therefore capillary pressure, is present in the evolution equation (35) and consequently influences the creep formulation, contradicts the ideal of an effective stress concept. According to the latter, all pore fluid pressure dependence of the mechanical response should be considered via the definition of the generalized effective stress only. Furthermore, there are a number of practical drawbacks associated with this approach. Since the effective stress is chosen as the only creep driving variable, long-term creep phenomena can be expected to be prominent in the simulation of drying shrinkage. In this approach, there is an influence of the viscous flow creep parameters $c_{0}$, $c_{1}$ and $q_{4}$ on $a_{\chi }$ and $b_{\chi }$. Since viscous flow creep is assumed to be driven by the effective stress, the predicted shrinkage strain does not approach an ultimate value. The calibration depends rather on the choice of a suitable time period. In [7], for instance, this time period was selected such that a good approximation to the measured strain was obtained at the time when the experiments were stopped.

#### 3.4.2. Multiphase Shrinkage and Creep Formulation in Terms of the Mixed Stress Concept

**Figure 2:**
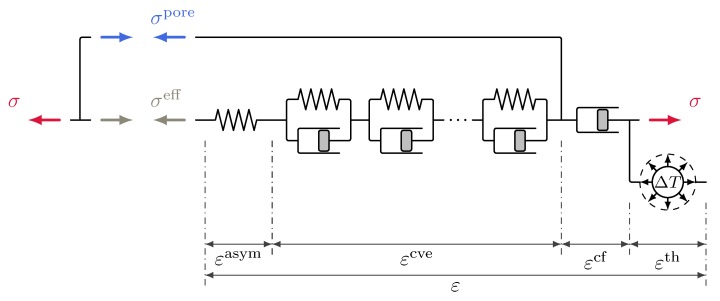
Schematic representation of the multiphase shrinkage and creep formulation in terms of the mixed stress concept; asymptotic elastic response and viscoelastic creep are driven by effective stress, viscous flow creep by total stress.

The concept of the mixed stress formulation is depicted in Figure 2. It is based on the idea of two different creep driving mechanisms in the formulation of the creep model, one for short-term (viscoelastic) and one for long-term (viscous flow) creep. In the mixed stress formulation these mechanisms are associated with two separate creep driving stress variables:

(45) $$\sigma^{\mathrm{drive},\;\mathrm{ve}}=\sigma^{\mathrm{eff}}\;\mathrm{and}\;\sigma^{\mathrm{drive},\;f}=\sigma \;.$$

The use of two separate creep driving stresses for these different mechanisms can also be identified in a paper by Hilaire et al. [34]. Therein, a thermo-chemo-mechanical model for basic creep under compressive and tensile loading is introduced. The stress variable driving viscoelastic creep in [34] is the total stress, while the viscous flow creep is driven by a modified version of the total stress. For the latter, the tensile components are scaled by an amplifying factor to account for micro-cracking. This allows modelling of different responses in compressive and tensile loading. Hilaire et al. [34] argue that the different mechanisms are related to different physical processes. This argument is adopted here for the fully coupled hygro-thermo-chemo-mechanical context, however with a different physical meaning and using different driving stresses (45). The first mechanism is a short-term micro-diffusion process. It is represented in the mixed stress formulation by the viscoelastic (solidification) part of the model driven by the effective stress. In that respect, the shrinkage formulation is the same as in the effective stress formulation described above. The second process is a shear slip mechanism associated with the breakage and reforming of C-S-H bonds, see also [30]. It is modelled by a viscous, long-term creep mechanism. In the mixed stress formulation, in contrast to the original model proposed by Gawin et al. [1,2], viscous flow creep is assumed to be driven by total stress. In the present model the influence of capillary pressure on the viscous flow part is considered in the evolution law (35) for the microprestress only, but not additionally via a viscous flow creep driving stress depending on capillary pressure.

The mixed stress approach is still completely consistent with respect to the shrinkage formulation in terms of the effective stress. Equations (42) and (44) still hold without further modification. Furthermore, the approach will allow to come up with tensile creep formulations in analogy to Hilaire et al. [34]. For the mixed stress approach uniform drying in the absence of external restraints can cause viscoelastic creep, but not viscous flow creep. Therefore, the predicted shrinkage strain eventually does approach an ultimate value, i.e., autogenous and drying shrinkage are bounded. The calibration process is also simplified significantly by the fact that the calibration of $a_{\chi }$ and $b_{\chi }$ is now independent of the long-term creep parameters.

#### 3.4.3. Multiphase Shrinkage and Creep Formulation in Terms of Total Stress

**Figure 3:**
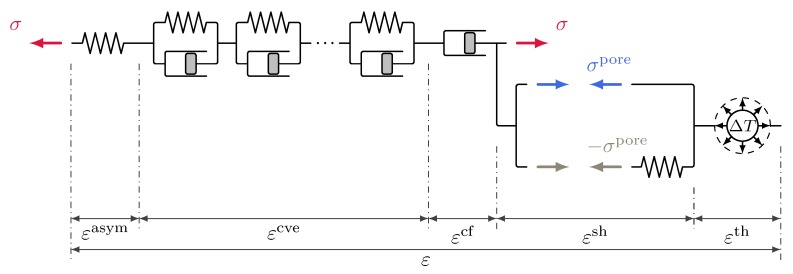
Schematic representation of the multiphase shrinkage and creep formulation in terms of total stress; viscous flow and viscoelastic creep are driven by total stress, shrinkage by the pore fluid stress (24).

In this formulation, schematically depicted in Figure 3, creep is assumed to be driven by the total stress only, viz.

(46) $$\sigma^{\mathrm{drive},\;\mathrm{ve}}=\sigma^{\mathrm{drive},\;f}=\sigma \;.$$

The stress-strain relationship is defined as

(47) $$\Delta \sigma =E_{\mathrm{qe}}\;\cdot \;G^{-1}\;\cdot \;\left(\Delta \varepsilon -\Delta \varepsilon^{\mathrm{cr}}-\Delta \varepsilon^{\mathrm{th}}-\Delta \varepsilon^{\mathrm{sh}}\right)\;\mathrm{with}\;\Delta \varepsilon^{\mathrm{sh}}=-1\;\cdot \;\frac{\Delta \left(\alpha_{\mathrm{Biot}}\;\cdot \;\chi \left(S_{w}\right)\;\cdot \;p^{c}\right)}{3K_{e}}\;.$$

Unlike for the other approaches, the shrinkage formulation in the stress-strain relation (47) is based on a rate-independent bulk modulus $K_{e}$ instead of a rate-dependent bulk modulus $K_{\mathrm{qe}}$ according to (43). Although being rate-independent, $K_{e}$ is time dependent since it is a function of the degree of hydration. In the present case it is chosen equal to the effective elastic-viscoelastic compression modulus $K_{\mathrm{qe}}$ evaluated for a fixed time step $\Delta t_{\mathrm{sh}}=1\mathrm{day}$, which is chosen to be on a time scale suitable for a shrinkage/drying process (i.e., rather on a time scale of days than seconds). Note that for this approach identical results can be generated for different choices of $\Delta t_{\mathrm{sh}}$ if the Bishop parameter is recalibrated. In this approach, creep will be present in a drying specimen without any external load only due to the presence of stresses due to restraint shrinkage caused by inhomogeneous drying. Such effects are present for instance during drying of cylindrical specimens investigated later in this paper. Due to the finite dimensions of the sample, restraint shrinkage will cause non-negligible total stresses inside the specimen which in turn will cause creep also in this formulation. For thin concrete slices however, as they are used for calibrating the Bishop parameter, the evolution of the creep deformation in this approach only reflects the change in capillary pressure due to drying. The formulation in terms of total stress shares most of the advantages of the formulation in terms of the mixed stress concept described in the previous subsection. However, in contrast to the effective and mixed stress formulations, a separate mechanical model is required for describing shrinkage. This separate mechanical model is assumed to be elastic with a stiffness derived from the elastic-viscoelastic part of the creep model. It is schematically depicted in Figure 3 by the additional spring element loaded by the pore fluid stress.

## 4. Comparison of the Shrinkage and Creep Formulations

In Section 4.1, the two shrinkage parameters $a_{\chi }$ and $b_{\chi }$ are calibrated based on drying shrinkage data obtained from thin concrete slices. The respective results are used to highlight fundamental characteristics of the three approaches. SubSection 4.2 is dedicated to a comparison of the three coupled shrinkage and creep formulations based on simulations of cylindrical specimens and comparison to experimental data from [8,9]. All simulations are performed using an in-house finite element software. This object-oriented software implements multiphase finite element formulations and multiphase materials for various applications in geotechnical and structural engineering. Discretization of the multiphase problem described in Section 2 results in a coupled system of nonlinear equations which is solved using Newton’s method. Efficient iterative solutions for the linear system in each Newton step are performed using a field-based block Gauss-Seidel preconditioning strategy with algebraic multigrid preconditioners applied for the approximate inversion of the diagonal blocks. More details on the implementation can be found in [35].

### 4.1. Drying Shrinkage of Thin Concrete Slices

The Bishop parameter for the three shrinkage formulations is calibrated based on experimental data obtained from drying of thin concrete slices with dimensions of 110 mm × 110 mm × 20 mm. As described in [9], these thin concrete slices were wet-stored up to the concrete age of 43 days, and subsequently exposed to drying in desiccators at 43%, 59%, 75%, and 85% relative humidity. In these experiments, mass and shrinkage strain were recorded for the thin concrete slices until the mass of the respective specimens attained a constant value. For calibrating the Bishop parameter, simulations are set up for each of the four levels of relative humidity. Each simulation begins at a concrete age of 8 h and considers the above-mentioned preconditioning process up to the concrete age of 43 days. Subsequently, the relative humidity is lowered to the level in the respective desiccator using the convective boundary condition (18). The evolution of the computed in-plane normal strain is compared to the experimental results. The two parameters $a_{\chi }$ and $b_{\chi }$ are adjusted until a sufficient agreement between experimentally observed and numerically predicted evolution of the shrinkage strain is achieved simultaneously for all four levels of relative humidity. Transport parameters governing the mass water content evolution in the slices are not altered during the calibration of $a_{\chi }$ and $b_{\chi }$. The calibration for the original effective stress formulation was performed in [7]. The result is used here for comparison to the other two formulations. The calibrated parameters for the three formulations are summarized in Table 9.

**Figure 4:**
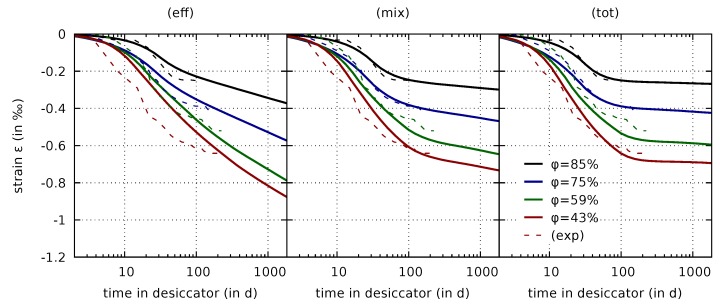
Drying shrinkage strain measured on thin slices [7,8] (exp) for different values of relative humidity φ and comparison to computed results on the basis of the three shrinkage and creep formulations: Effective stress formulation (eff), mixed stress formulation (mix), and total stress formulation (tot).

The predicted evolution of the drying shrinkage strain on the basis of the calibrated parameters are presented in Figure 4 together with the respective experimental data.

**Figure 5:**
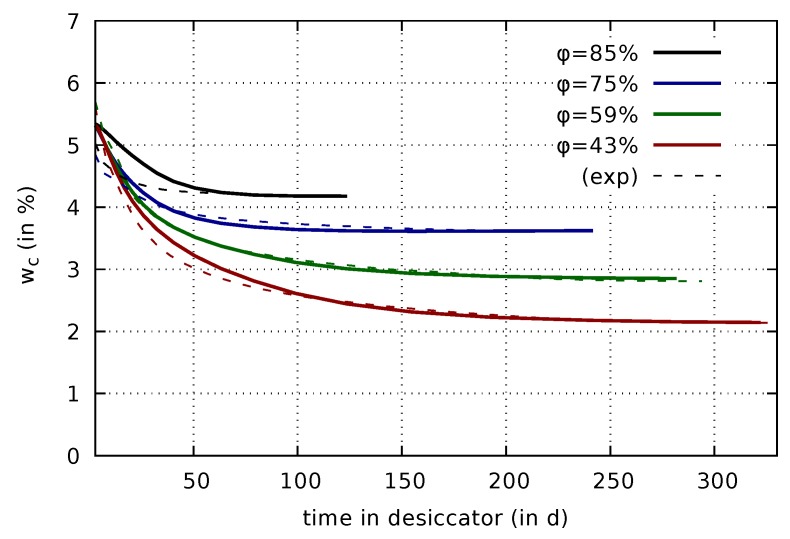
Computed and measured (exp) evolution of the mass water content $w_{c}$ in thin concrete slices ($w_{c}$ given with respect to the mass of the dry specimen).

They reveal significant differences between the three formulations. A considerably increasing drying shrinkage strain is predicted by the effective stress formulation, even years after the onset of drying, which is in contrast to the experimental data. It is emphasized that the computed water content evolution of the respective slices clearly approaches a limit value as shown in Figure 5. Hence, the unlimited increase of the computed strain is solely caused by the effective stress formulation.

A smaller amount of continuously increasing strain is visible also for the mixed stress formulation due to the viscoelastic part depending on the effective stress. However, it is much less pronounced than for the original formulation, and it is within the range of the experimental data. The results based on the mixed stress approach clearly exhibit the same S-type shape with a change in curvature in the semi-logarithmic plot as the experimental results. For the first 100 days, the results of the total stress and mixed stress formulation are rather similar. After the mass water content had approached a constant value, and, thus, drying had stopped, the total stress formulation provides almost constant values for the shrinkage strain. The small changes which are visible in Figure 4 (right) for long times in the desiccators are related to changing desorption isotherm properties, which evolve due to the still ongoing hydration process, rather than to the shrinkage and creep formulation, see [7] for more detailed information on the porosity-dependent desorption isotherm. In summary, the predicted strain evolution for both, the mixed and total stress formulation are much closer to the experimental data than the results obtained for the effective stress formulation. It is therefore much more straightforward to calibrate the shrinkage parameters for the mixed stress formulation, and similarly for the total stress formulation.

### 4.2. Shrinkage and Creep Tests of Cylindrical Specimens

Predictions on the basis of the shrinkage and creep formulations are compared with experimental results of shrinkage and creep tests on sealed and unsealed cylindrical specimens with 150 mm diameter and 450 mm height [8,9]. The ambient temperature is 293 K, and the ambient relative humidity is equal to 65%. The results for the effective stress formulation for the concrete age of up to 1000 days have been previously published in [7]. They will be used in the present subsection for comparison. Furthermore, a comparison of the experimental data and the simulation results for the mass water content evolution in the sealed and drying specimens is provided in [7]. Since the predicted mass water content evolution is not affected by the actual choice of the shrinkage and creep formulation, the respective results are equally valid for all three formulations.

#### 4.2.1. Prediction of Creep Compliance Functions

**Figure 6:**
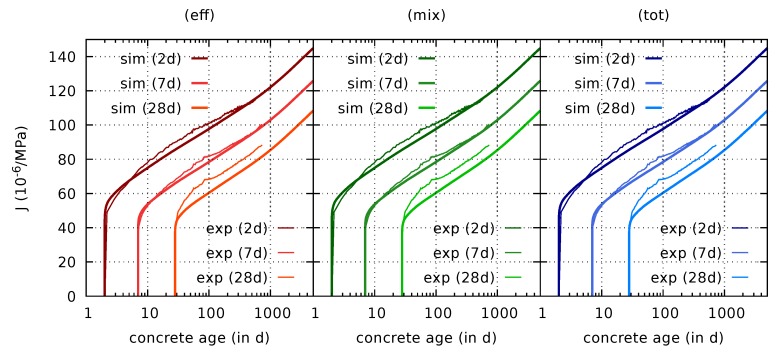
Computed (sim) and measured [8,9] (exp) creep compliance functions for sealed cylinders loaded at concrete ages of 2 days, 7 days, and 28 days, respectively, based on the effective stress formulation (eff), the mixed stress formulation (mix), and the total stress formulation (tot).

As pointed out in Section 3.2.1 and Section 3.2.2, the model predictions for the creep compliance functions are not affected by the choice of the creep driving stress. To illustrate this fact, results are presented for numerically obtained creep compliance functions for sealed cylinders loaded at concrete ages of 2 days to 5.49 MPa, 7 days to 8.7 MPa, and 28 days to 10.77 MPa, respectively. The loads correspond to 30% of the uniaxial compressive strength at the time of loading. The results are shown in Figure 6. They clearly prove the correctness of this claim.

Although not shown here, similar results are obtained for creep compliance functions for loaded drying specimens, as well as for the evolution of the elastic modulus. The respective figures are omitted here; they are identical with the results presented in [7].

#### 4.2.2. Prediction of Autogenous Shrinkage

**Figure 7:**
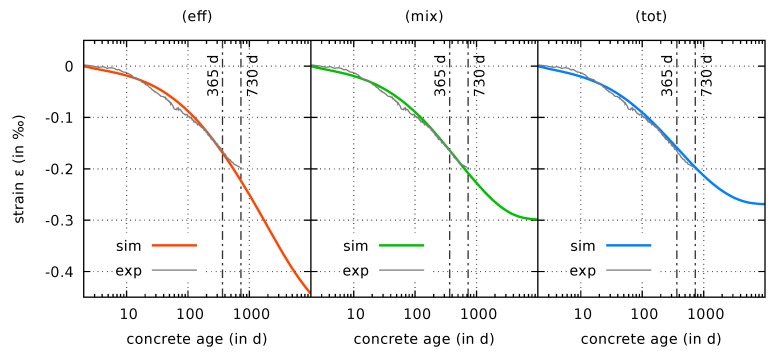
Computed (sim) and measured [8,9] (exp) evolution of the autogenous shrinkage strain based on the effective stress formulation (eff), the mixed stress formulation (mix), and the total stress formulation (tot).

The different coupled shrinkage and creep formulations are compared based on results of an autogenous shrinkage test performed on a sealed specimen [8,9]. The comparison of the computed autogenous shrinkage strains with the respective experimental data is shown in Figure 7, starting at the concrete age of two days.

The autogenous shrinkage strains are driven by the capillary pressure induced solely by internal desiccation due to cement hydration. The parameters describing the hydration process were calibrated in [7]. Excellent results were obtained not only for the hydration-induced temperature evolution in a calorimetry test, but also for the evolution of the mass water content in a sealed sample as well as for the hydration-dependent evolution of the Young’s modulus and the compressive strength. It is therefore concluded that the evolution of capillary pressure, which is driving the autogenous shrinkage deformation, is well predicted by the coupled model.

It can be seen in Figure 7 that all three formulations discussed in the present publication work well for predicting the evolution of the autogenous shrinkage strain for concrete ages of up to one year. However, for concrete ages exceeding one year, the predicted responses are characterized by increasing deviations from each other. On the one hand, the predicted autogenous shrinkage strain on the basis of the effective stress formulation keeps growing at a logarithmic rate, even beyond a concrete age of 1000 days. On the other hand, both the mixed and the total stress formulation, predict a progressively reduced growth rate for the autogenous shrinkage strain. For concrete ages ranging from one year up to two years, the predicted results for both the mixed stress formulation and the total stress formulation, are closer to the experimental data than the results obtained for the effective stress formulation. Furthermore, in contrast to the effective stress formulation, they both approach a limit value for the autogenous shrinkage strain, which is in the order of 0.3‰.

In the following, the results will be discussed in view of the findings presented in a recent work by Aili et al. [36]. Therein, the authors investigated how autogenous shrinkage of concrete evolves with time based on an analysis of experimental data from the database of Northwestern University [37]. The average test duration of the selected shrinkage data for sealed samples used in [36] is about half a year, whereas the longest measurement period is the one from the experiment by Mazloom et al. [38], providing data up to the concrete age of 586 days. Aili et al. [36] conclude that the autogenous shrinkage strain evolves logarithmically with respect to time in the long term. For concrete ages beyond 28 days, they fit the following empirical relation to the autogenous shrinkage strain data:

(48) $$\varepsilon_{\mathrm{fit}}\left(t\right)=\alpha_{\mathrm{fit}}\;\cdot \;\ln\left(\frac{t}{1\mathrm{day}}\right)+\beta_{\mathrm{fit}}\;.$$

**Figure 8:**
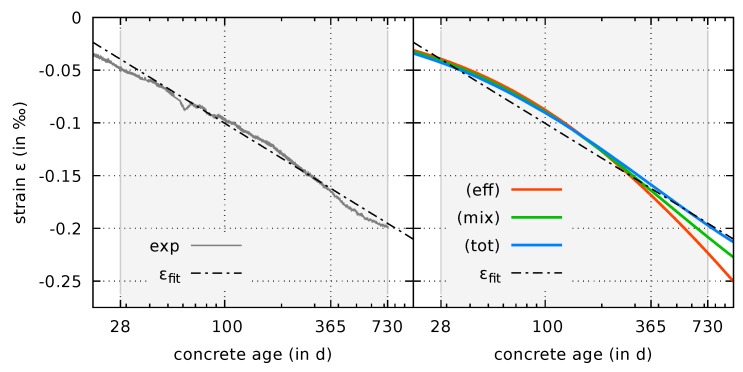
Fitted logarithmic approximation to experimental data from [8,9] (**left**) and comparison to computed evolutions of the autogenous shrinkage strain (**right**). The grey background color indicates the concrete age interval used for fitting.

At first view, the logarithmic evolution postulated by Aili et al. [36] based on the database analysis seems to be incompatible with the bounded ultimate shrinkage strain inherent to the mixed stress and total stress formulations. Therefore, the computed results will be discussed further with that respect. In a first step, a fit of the empirical relation (48) to the data from [8,9] is performed. The values $\alpha_{\mathrm{fit}}=-0.0477$, $\beta_{\mathrm{fit}}=0.119$, which are used in Figure 8, provide a fit which is a good approximation to the experimental data for concrete ages beyond 28 day, i.e., the logarithmic evolution is clearly present in the experimental data in the considered time interval.

Among the coupled shrinkage and creep formulations, the effective stress formulation is the only variant which predicts unlimited logarithmic growth of the autogenous shrinkage strain in the long term. The growth rate is controlled by the viscous flow creep parameters calibrated based on creep compliances in [7]. However, for concrete ages beyond one year the rate observed in Figure 7 (eff) is too high for the fitted empirical relation shown in Figure 8. Although the mixed and the total stress formulation do not evolve logarithmically with respect to time in the long term, it is shown in Figure 8 that they are very close to the fitted empirical relation in the time interval between 28 days and 730 days. Therefore, the results shown in Figure 8 indicate that all formulations are compatible with the logarithmic empirical evolution law postulated by Aili et al. [36] for concrete ages up to 730 days. The available experimental data does not allow a comparison beyond that age.

#### 4.2.3. Predictions of Drying Shrinkage of Cylindrical Specimens

**Figure 9:**
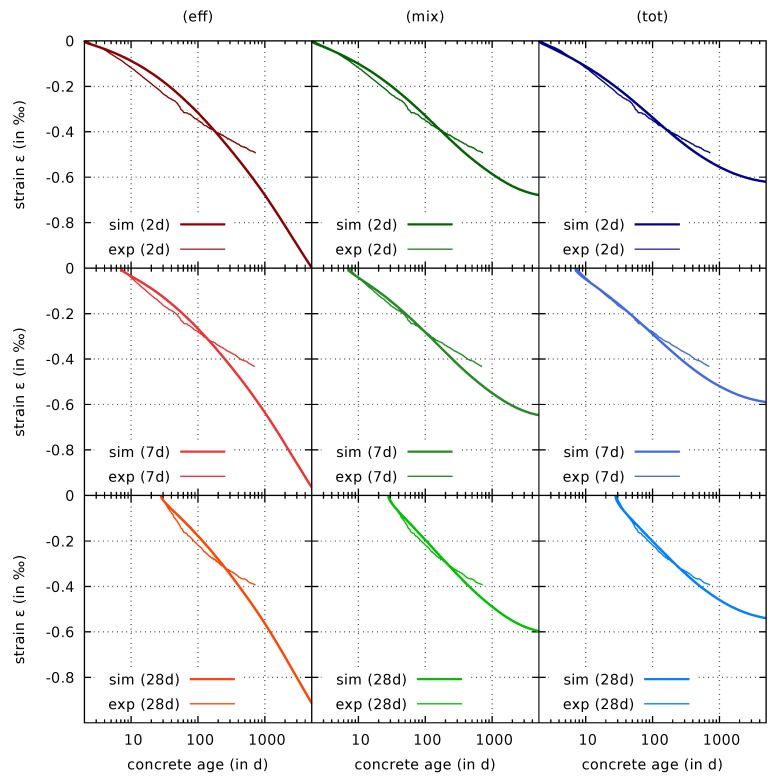
Computed and measured [8,9] evolution of the drying shrinkage for drying started at concrete ages of 2 days (first row), 7 days (second row), and 28 days (third row), respectively, for shrinkage formulations based on effective stress (eff), mixed stress (mix), and total stress (tot).

Figure 9 shows a comparison of predictions of the drying shrinkage response with the respective experimental data for three specimens, which were exposed to drying at concrete ages of 2 days, 7 days, and 28 days, respectively.

For the effective stress formulation, the results reveal a rapid growth of the drying shrinkage strain for long drying times. This behavior is caused by the viscous flow creep part of the model. The response is similar to the one for simulations of drying of the thin concrete slices in Figure 4. However, the concrete slices already attained hygral equilibrium within the first year of drying, whereas drying of the cylinders still continues beyond two years, and therefore increasing shrinkage strains can be expected. Although the experimental data clearly indicates a decreasing slope of the shrinkage strain curves in the semi-logarithmic plot for long drying times, the numerical results obtained with the effective stress formulation show a quite different trend. Unfortunately, the experimental data is limited to two years of drying. However, the results by Brooks [39] obtained from multi-decade drying shrinkage tests for other concrete mixtures confirm that the predicted continuing strain increase is rather unrealistic.

The predicted response is rather different for the mixed stress and total stress formulation. Although the strains after two years of drying are still somewhat overestimated, the appropriate trend is represented by approaching an ultimate drying shrinkage strain value. The predictions based on the mixed stress and the total stress formulation even outperform the effective stress formulation within the first year.

## 5. Conclusions

In this paper alternative coupled shrinkage and creep formulations for hygro-thermo-chemo-mechanical modelling of hardening concrete are presented. The major contributions of this work are:

1. Two new variants of a multiphase shrinkage and creep formulation are presented and are compared to the effective stress formulation by Gawin et al. [1,2]. The new formulations, termed mixed stress formulation and total stress formulation, differ from the original approach in the choice of the stress variable which drives viscous flow creep. The mixed stress formulation and the total stress formulation are based on different stress variables driving viscoelastic creep.
2. The effective stress formulation predicts viscoelastic and viscous flow creep due to drying. The mixed stress formulation predicts only viscoelastic creep due to drying. For the total stress formulation, creep occurs in drying specimens only as a (minor) secondary effect related to restraint stresses resulting from inhomogeneous drying.
3. The original effective stress formulation predicts significant shrinkage rates even after several years of autogenous shrinkage and drying shrinkage. Data from long-term shrinkage tests from the literature indicate that such a behavior contradicts test data. In contrast, the predicted shrinkage strains for both the mixed stress formulation and the total stress formulation, approach an ultimate value in the long term.
4. The new coupled shrinkage and creep formulations are calibrated based on drying shrinkage data from experiments on thin concrete slices. Improved predictions for the evolution of these drying shrinkage strains are obtained for both the mixed and total stress formulation.
5. For the new mixed and total stress formulation, the calibration of the shrinkage parameters is independent of the calibration of the long-term viscous flow creep parameters. Unlike in the original approach, shrinkage is decoupled from viscous long-term flow creep and vice versa.
6. The predictions of the evolution of the shrinkage strain for sealed and unsealed cylindrical specimens are significantly improved by the mixed and the total stress formulation. It is emphasized that only the generalized Bishop parameter is recalibrated for the different formulations based on the data from thin concrete slices. All other parts of the model, and its parameters determined in [7], remain unchanged. The improved predictions for the cylindrical specimens are therefore the consequence of the modification of the coupled shrinkage and creep formulation.

In summary, the new mixed stress formulation proves to be the most promising approach. It does not exhibit the severe overestimation of the long-term shrinkage strain which is typical for the original formulation. It furthermore decouples shrinkage and long-term viscous flow creep, a fact which significantly simplifies the calibration of the model. For the cylindrical specimens, the responses obtained with the mixed and total stress formulation are similar. However, in contrast to the total stress formulation, the mixed stress formulation has the advantage of a more natural definition of the effective elastic-viscoelastic bulk modulus.

## Figures and Tables

**Table 1:** Composition of the concrete mixture (concrete grade C30/37).

Component	Amount
Cement CEM II A-M (S-L) 42.5 N, *Lafarge*	375 $kg/m^{3}$
Added water (water/cement ratio of 0.44)	165 $kg/m^{3}$
Limestone sand 0/4 mm	810 $kg/m^{3}$
Limestone aggregates 4/8 mm	183 $kg/m^{3}$
Limestone aggregates 8/16 mm	457 $kg/m^{3}$
Limestone aggregates 16/32 mm	367 $kg/m^{3}$
Plasticizer *Proplast 200*	0.6% of cement mass
Air-entraining agent *Proair NVX*	0.045% of cement mass

**Table 2:** Parameters for the Kelvin-Laplace, Clausius Clapeyron, and ideal gas equations.

Parameter	Symbol	Value
Molar mass of air	$M_{a}$	$28.97\times 10^{-3}$ kg/mol
Molar mass of water	$M_{w}$	$18.01\times 10^{-3}$ kg/mol
Universal gas constant	*R*	8.314 J/(K mol)
Atmospheric pressure	$p^{\mathrm{atm}}$	$1013.25\times 10^{2}$ Pa
Water density	$\rho_{w}$	998.2 $kg/m^{3}$
Enthalpy of evaporation	$\Delta H_{\mathrm{vap}}$	$2.486\times 10^{6}$J/kg
Vapor pressure at full saturation at 293 K	$p_{\mathrm{sat},0}^{\mathrm{gw}}$	$2.3\times 10^{3}$ Pa

**Table 3:** Parameters for the hydration model and the porosity evolution [7].

Parameter	Symbol	Value
General parameter for Equation (7)	$A_{1}$	$1.35\times 10^{4}$/s
Parameter in Equation (7) governing the early-age response	$A_{2}$	$1.0\times 10^{-5}$
Parameter in Equation (7) governing the long-term response	$\bar{\eta }$	11.0
Estimated ultimate degree of hydration according to [20]	$\kappa_{\infty }^{w/c}$	0.716
Activation energy (of lumped model) divided by the universal gas constant	$E_{a}/R$	5000 K
Degree of hydration at which concrete is assumed to solidify	$\Gamma_{\mathrm{init}}$	0.1
Ultimate amount of chemically bound water per unit volume	$\Delta m_{w}^{\infty }$	63.8 $kg/m^{3}$
Solid phase density at 293 K	$\rho_{s}$	2606 $kg/m^{3}$
Porosity at full hydration	$n_{\infty }$	13.89%
Parameter governing the decrease of porosity with increasing hydration	$A_{n}$	2.45%

**Table 4:** Parameters for the desorption isotherm [7].

Parameter	Symbol	Value
First van Genuchten parameter	$n_{\mathrm{Sw}}$	0.6
Second van Genuchten parameter	$m_{\mathrm{Sw}}$	0.185
Dimensionless fitting parameter	*D*	0.225
Parameter for porosity-dependence of the air entry value	ψ	27.0
Air entry value at full hydration	$p_{b}^{c}\left(n_{\infty }\right)$	4.6 MPa

**Table 5:** Parameters for water transport [7].

Parameter	Symbol	Value
Asymptotic intrinsic permeability at full hydration	$K_{\infty }$	$1.45\times 10^{-22}$ m^2^
Parameter for hydration dependency of the permeability	$A_{\mathrm{perm}}$	2.5
Van Genuchten-Mualem parameter in Equation (16)	$m_{\mathrm{kw}}$	0.43
Dynamic viscosity of water	$\mu_{w}$	$9.94\times 10^{-4}$ Pa s
Coefficient of diffusion in the air at reference temperature	$D_{g,0}^{\mathrm{gw}}$	$2.58\times 10^{-5}$ m^2^/s
Exponent porosity dependence of resistance factor	$a_{\mathrm{fS}}$	2.0
Exponent saturation dependence of resistance factor	$b_{\mathrm{fS}}$	4.5
Convective mass transport coefficient (on boundary)	$\beta_{W}$	$5\times 10^{-14}$kg/(s m^2^)

**Table 6:** Parameters for the enthalpy balance [7].

Parameter	Symbol	Value
Specific heat capacity of water	$C_{p}^{w}$	$4.182\times 10^{3}$J/(kgK)
Specific heat capacity of dry air	$C_{p}^{\mathrm{ga}}$	$1.012\times 10^{3}$J/(kgK)
Specific heat capacity of the solid phase	$C_{p}^{s}$	$0.855\times 10^{3}$J/(kgK)
Effective heat conductivity of concrete	$\lambda_{\mathrm{eff}}$	1.5W/(mK)
Released heat of hydration per unit volume	$Q_{\infty }$	$190\times 10^{6}$J/$m^{3}$
Convective heat transfer coefficient	$\beta_{T}$	9 W/($m^{2}$ K)

**Table 7:** Parameters for the creep formulation [7].

Parameter	Symbol	Value
Poisson’s ratio	ν	0.2
Smallest retardation time in Dirichlet series	$\tau_{0}^{\mathrm{Kelvin}}$	$1\times 10^{-5}$ day
Number of Kelvin units in Dirichlet expansion	*N*	8
Power-law exponent for microscopic creep compliance function	$n^{\mathrm{Kelvin}}$	0.1
Viscoelastic compliance parameter	$q_{2}$	$27\times 10^{-6}$/MPa
Viscous flow creep parameter	$q_{4}$	$7.9\times 10^{-6}$/MPa
Initial value of the microsprestress at 1 day	$S_{0}$	625 MPa
Parameter for evolution of microprestress	$c_{0}$	$1.6\times 10^{-3}$/(MPa day)
Parameter for dependence of evolution of microprestress on humidity changes	$c_{1}$	10 MPa

**Table 8:** Parameters for the incremental stress-strain relationship and thermal expansion [7].

Parameter	Symbol	Value
Asymptotic Young’s modulus at full hydration	$E_{\mathrm{asym},\;\infty }$	70,180 MPa
Power-law exponent for asymptotic Young’s modulus	$b_{E}$	0.16
Thermal expansion coefficient	$\alpha_{T}$	$1.1\times 10^{-5}$/K

**Table 9:** Calibrated values for the generalized Bishop parameter in equation (25) for the original effective stress formulation (eff), for the mixed stress formulation (mix), and for the total stress formulation (tot).

Parameter	Symbol	(eff)	(mix)	(tot)
First parameter in (25)	$a_{\chi }$	0.67	0.75	0.9
Second (offset) parameter in (25)	$b_{\chi }$	0.08	0.04	0.02

## References

[1] Gawin D., Pesavento F., Schrefler B.A. (2006). Hygro-thermo-chemo-mechanical modelling of concrete at early ages and beyond. Part I: hydration and hygro-thermal phenomena. Int. J. Numer. Meth. Eng..

[2] Gawin D., Pesavento F., Schrefler B.A. (2006). Hygro-thermo-chemo-mechanical modelling of concrete at early ages and beyond. Part II: shrinkage and creep of concrete. Int. J. Numer. Meth. Eng..

[3] Sciumè G., Benboudjema F., De Sa C., Pesavento F., Berthaud Y., Schrefler B.A. (2013). A multiphysics model for concrete at early age applied to repairs problems. Eng. Struct..

[4] Valentini B., Theiner Y., Aschaber M., Lehar H., Hofstetter G. (2013). Single-phase and multi-phase modeling of concrete structures. Eng. Struct..

[5] Neuner M., Gamnitzer P., Hofstetter G. (2017). An extended damage plasticity model for shotcrete: Formulation and comparison with other shotcrete models. Materials.

[6] Gasch T., Eriksson D., Ansell A. (2019). On the behaviour of concrete at early-ages: A multiphase description of hygro-thermo-chemo-mechanical properties. Cem. Concr. Res..

[7] Gamnitzer P., Drexel M., Brugger A., Hofstetter G. (2019). Calibration of a multiphase model based on a comprehensive data set for a normal strength concrete. Materials.

[8] Theiner Y., Drexel M., Neuner M., Hofstetter G. (2017). Comprehensive study of concrete creep, shrinkage, and water content evolution under sealed and drying conditions. Strain.

[9] Drexel M., Theiner Y., Hofstetter G. (2018). Versuche zum Schwinden und Kriechen von Beton unter Berücksichtigung des Feuchtegehalts. Bauingenieur.

[10] Drexel M., Theiner Y., Hofstetter G. (2018). Investigation of tensile creep of a normal strength overlay concrete. Materials.

[11] Drexel M., Smaniotto S., Hofstetter G. (2019). Complementary experimental study of a normal strength overlay concrete. Mater. Today-Proc..

[12] Alonso E.E., Gens A., Josa A. (1990). A constitutive model for partially saturated soils. Géotechnique.

[13] Kohler R., Hofstetter G. (2008). A cap model for partially saturated soils. Int. J. Numer. Anal. Meth. Geomech..

[14] Gamnitzer P., Hofstetter G. (2015). A smoothed cap model for variably saturated soils and its robust numerical implementation. Int. J. Numer. Anal. Meth. Geomech..

[15] Nuth M., Laloui L. (2008). Effective stress concept in unsaturated soils: Clarification and validation of a unified framework. Int. J. Numer. Anal. Meth. Geomech..

[16] Lewis R.W., Schrefler B.A. (1998). The Finite Element Method in the Static and Dynamic Deformation and Consolidation of Porous Media.

[17] Ulm F.-J., Coussy O. (1996). Strength growth as chemo-plastic hardening in early age concrete. J. Eng. Mech..

[18] Cervera M., Oliver J., Prato T. (1999). Thermo-chemo-mechanical model for concrete I: Hydration and aging. J. Eng. Mech..

[19] Torrenti J.M., Benboudjema F. (2005). Mechanical threshold of cementitious materials at early age. Mater. Struct..

[20] Pantazopoulou S.J., Mills R.H. (1995). Microstructural aspects of the mechanical response of plain concrete. ACI Mater. J..

[21] Bažant Z.P., Jirásek M. (2018). Creep and Hygrothermal Effects in Concrete Structures.

[22] Chitez A.S., Jefferson A.D. (2015). Porosity development in a thermo-hygral finite element model for cementitious materials. Cem. Concr. Res..

[23] Van Genuchten M.T. (1980). A closed-form equation for predicting the hydraulic conductivity of unsaturated soils. Soil Sci. Soc. Am. J..

[24] Gallipoli D., Wheeler S.J., Karstunen M. (2003). Modelling the variation of degree of saturation in a deformable unsaturated soil. Géotechnique.

[25] Gawin D., Pesavento F., Schrefler B.A. (2003). Modelling of hygro-thermal behaviour of concrete at high temperature with thermo-chemical and mechanical material degradation. Comput. Methods Appl. Mech. Eng..

[26] Mualem Y. (1976). A new model for predicting the hydraulic conductivity of unsaturated porous media. Water Resour. Res..

[27] Gawin D., Majorana C.E., Schrefler B.A. (1999). Numerical analysis of hygro-thermal behaviour and damage of concrete at high temperature. Mech. Cohes.-Frict. Mater..

[28] Bažant Z.P., Prasannan A. (1989). Solidification theory for concrete creep. I: Formulation. J. Eng. Mech..

[29] Carol I., Bažant Z.P. (1993). Viscoelasticity with aging caused by solidification of nonaging constituent. J. Eng. Mech..

[30] Bažant Z.P., Hauggaard A.B., Baweja S., Ulm F.-J. (1997). Microprestress-solidification theory for concrete creep. I: Aging and drying effects. J. Eng. Mech..

[31] Bažant Z.P., Hauggaard A.B., Baweja S. (1997). Microprestress-solidification theory for concrete creep. II: Algorithm and Verification. J. Eng. Mech..

[32] Bažant Z.P., Xi Y. (1995). Continuous retardation spectrum for solidification theory of concrete creep. J. Eng. Mech..

[33] Jirásek M., Havlásek P. (2014). Microprestress-solidification theory of concrete creep: Reformulation and improvement. Cem. Concr. Res..

[34] Hilaire A., Benboudjema F., Darquennes A., Berthaud Y., Nahas G. (2014). Modeling basic creep in concrete at early-age under compressive and tensile loading. Nucl. Eng. Des..

[35] Gamnitzer P., Hofstetter G. (2016). Fully coupled multi-phase modelling of pumping induced settlements, air- and water flow in multi-layered normally consolidated soils. Comput. Geotech..

[36] Aili A., Vandamme M., Torrenti J., Masson B. (2018). Is long-term autogenous shrinkage a creep phenomenon induced by capillary effects due to self-desiccation?. Cem. Concr. Res..

[37] Bažant Z.P., Li G.H. (2008). Comprehensive database on concrete creep and shrinkage. ACI Mater. J..

[38] Mazloom M., Ramezanianpoura A.A., Brooks J.J. (2004). Effect of silica fume on mechanical properties of high-strength concrete. Cem. Concr. Comp..

[39] Brooks J.J. (2005). 30-year creep and shrinkage of concrete. Mag. Concr. Res..

